# Tradeoff between User Experience and BCI Classification Accuracy with Frequency Modulated Steady-State Visual Evoked Potentials

**DOI:** 10.3389/fnhum.2017.00391

**Published:** 2017-07-26

**Authors:** Alexander M. Dreyer, Christoph S. Herrmann, Jochem W. Rieger

**Affiliations:** ^1^Applied Neurocognitive Psychology Laboratory, Department of Psychology, Center for Excellence “Hearing4all”, European Medical School, Carl von Ossietzky University Oldenburg, Germany; ^2^Experimental Psychology Laboratory, Department of Psychology, Center for Excellence “Hearing4all”, European Medical School, Carl von Ossietzky University Oldenburg, Germany; ^3^Research Center Neurosensory Science, Carl von Ossietzky University Oldenburg, Germany

**Keywords:** steady-state visual evoked potentials (SSVEP), frequency modulation, brain-computer interface (BCI), electroencephalography (EEG), spatial attention

## Abstract

Steady-state visual evoked potentials (SSVEPs) have been widely employed for the control of brain-computer interfaces (BCIs) because they are very robust, lead to high performance, and allow for a high number of commands. However, such flickering stimuli often also cause user discomfort and fatigue, especially when several light sources are used simultaneously. Different variations of SSVEP driving signals have been proposed to increase user comfort. Here, we investigate the suitability of frequency modulation of a high frequency carrier for SSVEP-BCIs. We compared BCI performance and user experience between frequency modulated (FM) and traditional sinusoidal (SIN) SSVEPs in an offline classification paradigm with four independently flickering light-emitting diodes which were overtly attended (fixated). While classification performance was slightly reduced with the FM stimuli, the user comfort was significantly increased. Comparing the SSVEPs for covert attention to the stimuli (without fixation) was not possible, as no reliable SSVEPs were evoked. Our results reveal that several, simultaneously flickering, light emitting diodes can be used to generate FM-SSVEPs with different frequencies and the resulting occipital electroencephalography (EEG) signals can be classified with high accuracy. While the performance we report could be further improved with adjusted stimuli and algorithms, we argue that the increased comfort is an important result and suggest the use of FM stimuli for future SSVEP-BCI applications.

## Introduction

Steady-state visual evoked potentials (SSVEPs) have been employed for the control of a wide variety of brain-computer interfaces (BCIs). SSVEPs are typically elicited by visual stimuli with periodically varying light intensities. The stimuli can be presented on a computer display (e.g., checkerboard patterns) or with flickering light-emitting diodes (LEDs). Such stimulus variations elicit periodic variations of electric potentials over the occipital cortex, measurable with electroencephalography (EEG), at the same frequency as the stimulus intensity variation plus harmonics thereof. The reliability of SSVEP responses to a large range of stimulation frequencies (Herrmann, 2001) allows, in principle, for a high number of possible BCI commands. Their high signal-to-noise ratio (SNR) leads to correct signal classifications within seconds which in turn leads to high information transfer rates (ITRs). For an extensive review of the SSVEP-BCI literature see Vialatte et al. (2010). While most previous studies have focused on increasing ITRs and classification accuracies of SSVEP-based BCIs, only few took the user perspective and tried to improve their experience with the BCI systems.

Despite being attractive from the technical perspective, SSVEP-based BCI systems have the important drawback that the use of several independently flickering light sources can rapidly produce user discomfort and fatigue. In fact, Cao et al. (2014) showed increasing levels of fatigue after SSVEP stimulation by using subjective as well as physiological measures and Ortner et al. (2011) report that their subjects disliked flickering light stimuli. Additionally, it has been shown that low frequency flicker is more perceptible and more irritating for the user (Lin et al., 2012). Stimulation with higher frequencies reduces discomfort (Sakurada et al., 2015) but decreases BCI performance and increases the rate of BCI illiteracy (Volosyak et al., 2011). These studies indicate that, in order to enhance the usability of SSVEP-based BCIs the traditionally used stimuli should be improved.

One way to improve stimuli for SSVEP-BCIs is to combine high frequency stimulation, which should be more comfortable for the user, with low SSVEP frequency, which improves classification rates. Indeed, Chang et al. (2014) reported a reduced sense of flicker and fatigue for amplitude modulated signals. We recently introduced frequency modulated (FM) stimuli (Dreyer and Herrmann, 2015) in which a high frequency carrier is modulated by a second frequency. The spectra of FM signals can be designed to have distinct peaks at the frequency of the difference of the carrier and the modulation frequency. For example a 100 Hz carrier modulated at a frequency of 90 Hz would elicit an SSVEP at 10 Hz (= 100 Hz − 90 Hz). We were able to show that FM stimuli with either low or high carrier frequencies elicited 10 Hz SSVEPs which did not differ significantly from SSVEPs elicited with a 10 Hz sinusoidal (SIN) stimulus. However, the subjective perceptibility of the flicker decreased with increasing FM carrier frequencies. This lead us to hypothesize that FM stimuli would be preferable for future SSVEP-BCI implementations.

To further explore the potential of FM stimuli for SSVEP-based BCIs, we had several goals in the current study. Firstly, we wanted to demonstrate that FM-SSVEPs can reliably be evoked in an extended frequency range between 20 Hz and 29 Hz with several independently flickering LEDs. While Dreyer and Herrmann (2015), using 10 Hz FM stimulation, might have evoked FM-SSVEPs solely by entrainment of the alpha oscillator, the current stimulation frequencies were chosen in a different band to show that alpha entrainment is not a necessity for evoking FM-SSVEPs.

Our second aim was to gather additional evidence for the FM stimulation being more comfortable on a behavioral level. The third aim was to compare the classification accuracies for FM-SSVEPs with SIN-SSVEPs to show the suitability of FM stimulation for BCI systems.

One further challenge for SSVEP-BCIs in general is the question of dependency on muscle control. A BCI depending on eye-movements could not be used by people who lost control over their gaze. Previous research showed modulations of SSVEP amplitudes by attention shifts which could be used to control a BCI (Kelly et al., 2004; Allison et al., 2010). As our fourth goal, we therefore wanted to test whether covert spatial attention shifts towards a specific LED would be sufficient to evoke and classify FM-SSVEP responses. Following classic attention research terminology (e.g., Posner, 1980) and similar to other researcher (Walter et al., 2012), we will use the term “overt attention” for the fixation of an LED by gaze shifts and the term “covert attention” for an attention shift towards an LED without eye movements.

## Materials and Methods

### Participants

We recorded EEG data from 13 participants (10 female) with a mean age of 22.8 years (range from 20 years to 25 years). All participants had normal or corrected-to-normal vision and were informed about the risk of seizures in epileptics due to flicker stimulation prior to the experiment. None of the participants reported to have ever suffered from epilepsy or other seizures. All participants gave written informed consent in accordance with the Declaration of Helsinki. The study was approved by the ethics committee of the University of Magdeburg (former affiliation of CSH and JWR). In addition, subjects were informed about muscle and movement artifacts in EEG signals and instructed to avoid movement during the stimulation.

### Stimuli

**Figure 1:**
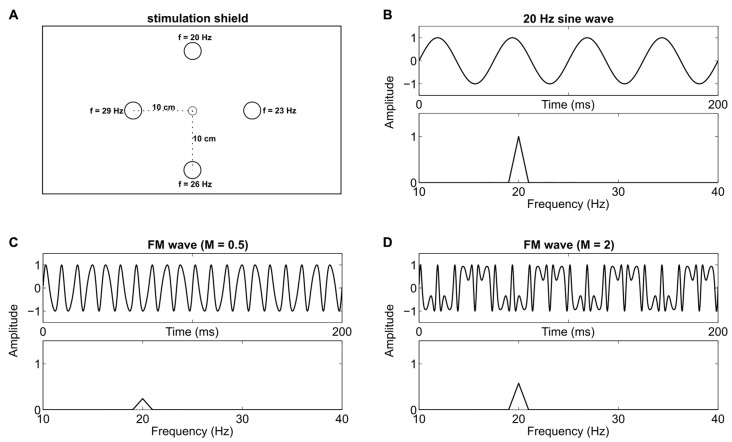
Stimulation device and signal overview. **(A)** Custom stimulation shield with a small central fixation light-emitting diode (LED) and four stimulation LEDs at 10 cm (5.72° visual angle at 1 m distance) radial distance. One specific Steady-state visual evoked potential (SSVEP) frequency was assigned to each stimulation LED throughout the experiment. **(B)** A 20 Hz sine wave which was used to drive one LED, and the respective frequency spectrum. **(C)** A frequency modulated (FM) signal (100 Hz carrier, 80 Hz modulation frequency and a modulation index of 0.5) and its frequency spectrum. **(D)** A signal with the same parameters as in **(C)** except that the modulation index is increased to 2. This leads to an increased peak in the spectra at 20 Hz.

We used five LEDs mounted on a 56 cm × 71 cm custom cardboard shield (see Figure 1A) for visual stimulation. One green LED (diameter 0.5 cm) served as central fixation target, and four green/red LEDs (diameter 1 cm) as stimulation targets, placed at 10 cm radial distance in the corners of a diamond. The distance between the central LED and the participants’ nasion was kept at approximately 1 m. Therefore, the size of the central fixation LED and the stimulation LEDs was about 0.29°/0.57° visual angle respectively. A digital-to-analog converter (NI USB-6229 BNC, National Instruments, Austin, TX, USA) with 16 bit resolution, 10 kHz sampling rate, and a custom signal amplifier were used to drive the LEDs at different frequencies. We generated SIN and FM signals with MATLAB (The MathWorks Inc., Natick, MA, USA) using the following formulas:

Sinusoidal modulation

(1) $$signal_{\text{SIN}}=A+FV*\sin\;(2*\pi *F*t),$$

FM sinusoidal carrier

(2) $$signal_{\text{FM}}=A+FV*\sin\;\{2*\pi *Fc*t+[M*\sin\;(2*\pi *Fm*t)]\}.$$

Here, *A* is the DC bias (2.5 V) at which the LED was driven and *FV* is the flicker voltage span (1.8 V). Numbers in parentheses state the values we used in the actual experiment. *F* represents the stimulation frequency for the SIN signals (20 Hz, 23 Hz, 26 Hz or 29 Hz). *Fc* (100 Hz) is the carrier frequency and *Fm* (80 Hz, 77 Hz, 74 Hz or 71 Hz) is the modulation frequency of the FM signal. *M* is the modulation index (2) and *t* represents ongoing time. Exemplary signal traces with arbitrary parameters can be seen in Figures 1B–D. The chosen voltages led to brightness changes of the LEDs between a light glimmer and their maximum possible brightness. The time course of the light signal was tested with a photodiode and an oscilloscope. Note that the differences between the carrier and the modulation frequencies match the stimulation frequencies we used for SIN stimulation. Therefore, SSVEP peaks should be evoked at these same frequencies (Dreyer and Herrmann, 2015) in both conditions. In the current study, we used *M* = 2, in contrast to *M* = 0.5 which was used by Dreyer and Herrmann (2015). The modulation index *M* is the ratio between the frequency deviation and the modulation frequency. Different *M* values change the distribution of signal power in the sidebands. We used *M* = 2 because, compared to *M* = 0.5, this increases the power of the target frequency in the stimulation signal (see Figures 1C,D). Throughout the experiment, each stimulation frequency was permanently assigned to one LED. The top LED evoked 20 Hz, the right LED 23 Hz, the bottom LED 26 Hz and the left LED 29 Hz SSVEPs (see Figure 1A).

### Data Acquisition and Experimental Procedure

Participants were comfortably seated in an electrically shielded recording chamber with dim ambient light. We used a Brain Amp EEG amplifier and Brain Vision Recorder (Brain Products GmbH, Gilching, Germany) for EEG acquisition. Sampling rate was set to 500 Hz and the amplifier’s frequency passband ranged from 0.1 Hz to 250 Hz. According to the international 10–10 system, 32 electrodes, including one vertical EOG electrode below the right eye, were placed on size-appropriate EEG caps. Another electrode placed on the nose was used as recording reference.

At the beginning of the session we included three habituation runs with around 90 s length. During the first, all stimulation LEDs were lit up statically (i.e., not flickering) at medium brightness (2.5 V). During the second and third, all LEDs flickered simultaneously with SIN or FM modulation respectively, exactly at the frequencies used during the experimental conditions. The subjects were instructed to look at the central fixation LED in all three habituation runs. This procedure allowed the subjects to adjust their vision to the relatively dark recording chamber and the flickering LEDs.

**Figure 2:**
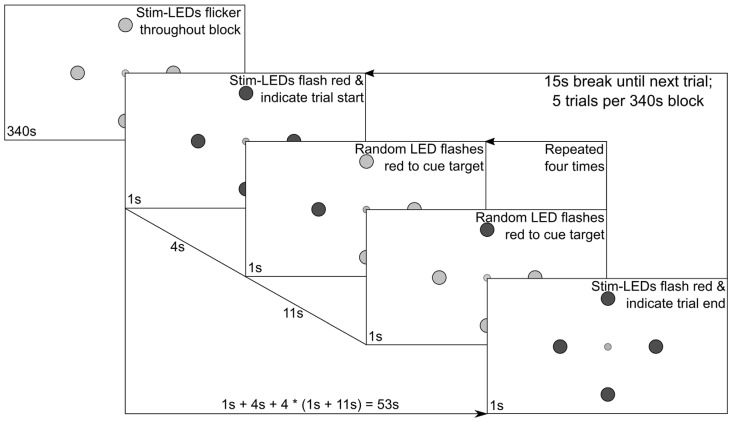
Experimental procedure. The central fixation LED was lit up at constant brightness and the surrounding stimulation LEDs flickered constantly at individual frequencies. Following the depicted protocol, intermittent red flashes of the stimulation LEDs were used to cue subject behavior.

Subsequently, eight experimental stimulation blocks were run. In the first four blocks, all LEDs flickered either with SIN or FM driving signals and the subjects were instructed to overtly shift their attention towards one specific LED following a visual cue. The order of these four blocks was pseudo-randomized across subjects, with the constraint that during the first two blocks the stimuli were always presented either with SIN or FM stimulation, i.e., the order was either (SIN, SIN, FM, FM) or (FM, FM, SIN, SIN). During the last four blocks the subjects were instructed to constantly fixate the central LED and to only covertly shift their spatial attention towards the cued LEDs. Only FM stimulation was used in these blocks. Figure 2 shows a diagram of the stimulation sequence for a single block which lasted 340 s.

Each block consisted of five trials. In the beginning of each trial, all four stimulation LEDs flashed red for 1 s to indicate the start of the trial. After another 4 s, a random stimulation LED flashed red for 1 s, signaling the subjects to redirect their gaze or their focus of attention on that position until another LED flashed red to indicate the next target position. This was repeated in a pseudo-randomized order until all four LEDs were overtly gazed at or covertly focused once. Between the red cues, all stimulation LEDs flickered green at their assigned frequency, i.e., the physical stimuli were equal, only the subjects’ gaze/attention changed. The end of a trial was again indicated by all LEDs flashing red. In total, each trial was 53 s long and followed by a 15 s break, giving the subjects time to rest their eyes. Between blocks, the subjects could decide how long the break should be which was in most cases not more than a minute. For each LED position, 55 s of stimulation were recorded per block, leading to 110 s of EEG data from the overt attention conditions (two blocks per condition) and 220 s of EEG data in the covert spatial attention condition (four blocks).

After each of the overt attention blocks, the subjects rated their comfort level during the stimulation on a 1-to-5 Likert scale ranging from 1—very uncomfortable to 5—very comfortable. These ratings were available from 12 subjects, as one of the authors of this study (AD) was among the subjects. His behavioral data was not recorded to avoid bias.

### EEG Data Preprocessing

We used MATLAB and functions from the EEGLAB toolbox (Delorme and Makeig, 2004) to process the EEG data. First, we checked standard deviations per channel and visually rejected bad channels with excessively high noise before re-referencing to a common-average. The data were high-pass filtered at 1 Hz, low-pass filtered at 200 Hz and notch-filtered between 49 Hz and 51 Hz to suppress power line noise. All filters were third order Butterworth filters applied with MATLAB’s “filtfilt” function. For further analysis, data from the two blocks per overt attention condition were combined. Equally, we combined the data from the four covert attention blocks.

SSVEP analysis was achieved by time-locking to the stimulation signals and cutting the data into 1 s epochs. We then calculated amplitude spectra of these epochs using the fast Fourier transform. One second windows were chosen to keep our results in that respect comparable to Dreyer and Herrmann (2015) which is so far the only article on FM-SSVEPs. It has been shown, that some subjects are not able to control SSVEP-BCIs, especially with high frequencies (Volosyak et al., 2011). As SSVEPs typically have a high SNR, we used a short reasonable epoch-length (1 s) to compare SSVEP amplitudes to identify such non-responders. When comparing SSVEP-amplitudes for different frequencies, it is important that the spectra are based on time-windows that contain an integer number of stimulation cycles (Bach and Meigen, 1999). Subjects that showed no apparent SSVEP peaks at the stimulation frequencies will be referred to as non-responders. They will be included in the classification analysis, but the results will be presented separately.

### Feature Generation and Classification

Approaches for generating SSVEP features often use power spectral density analysis (PSDA) or canonical correlation analysis (CCA; Lin et al., 2006). We used CCA which has been shown to perform better in detecting visual steady-state signals acquired with several imaging techniques (EEG: Hakvoort et al., 2011; magnetoencephalography: Reichert et al., 2013b). Another benefit of CCA is that it can be used as a training-independent classifier, thereby bypassing the need for model generation and cross-validation. Before applying CCA, we band-pass filtered the EEG data with cutoff frequencies at 15 Hz and 65 Hz. This retains signal variations in the dynamical range between the lowest target frequency (20 Hz) and the highest first harmonic frequency (58 Hz). The first harmonics were included in the analysis as they have been shown to contribute to SSVEP classification performance (Müller-Putz et al., 2005). Moreover, we tested different window lengths (1 s, 2 s,…, up to 11 s) and used data from several occipital and parietal electrodes, namely O1, O2, Pz, P3, P4, P7 and P8 for the CCA. These occipital channels were correlated via CCA with a sine and cosine wave at each of the respective stimulation frequencies as well as their first harmonics. For the four stimulation frequencies, the highest squared sum of the canonical correlation coefficients was used as the decision criterion. Classification accuracies and the respective window lengths were used to calculate ITRs as described in Wolpaw et al. (1998) using the following equation:

(3) $$ITR\;=\;\left[log_{2}(N)\;+\;p\;log_{2}(p)\;+\;(1-p)\;log_{2}\left(\frac{1-p}{N-1}\right)\right]*\frac{60s}{t_{e}}$$

Here *N* is the number of targets (4), *p* is the classification accuracy and *t_e_* is the window length used for classification in seconds. As opposed to proportion of correct classifications, the ITR considers both, the classification accuracy and the length of the analysis interval. It provides a measure (bits per minute) of how much information a BCI system could transfer in a certain period of time.

## Results

### Comfort Rating

Since high frequency stimulation tends to be less annoying (Lin et al., 2012) and FM stimulation with a high frequency carrier is less perceptible (Dreyer and Herrmann, 2015), our hypothesis was that FM stimulation would on average be more comfortable than SIN stimulation. We first compared the ratings in the two blocks for each condition separately with paired sample *t*-tests. There was neither a significant difference between the ratings of the SIN conditions (*M*_SIN1_ = 2.5, *SD*_SIN1_ = 0.9, *M*_SIN2_ = 2.5, *SD*_SIN2_ = 1; *t*_(11)_ = 0, *p* = 1) nor between the ratings of the FM conditions (*M*_FM1_ = 3.08, *SD*_FM1_ = 0.79; *M*_FM2_ = 2.83, *SD*_FM2_ = 0.72; *t*_(11)_ = 1.39, *p* > 0.1). Therefore, we combined the ratings over blocks to create one mean rating for the SIN and of the FM stimulation, respectively, per subject for further statistical analysis. Table 1 shows the individual ratings averaged over blocks. Testing our hypothesis, we used a one-sided, paired-sample *t*-test to compare the rating between the two stimulation conditions. There was a significant difference in the average rating of the SIN condition (*M* = 2.5, *SD* = 0.88) and the rating of the FM condition (*M* = 2.96, *SD* = 0.69; *t*_(11)_ = 3.19, *p* < 0.01). These results suggest that FM stimulation is indeed perceived as more comfortable. Although the mean difference is small, the effect is very consistent over subjects as only 1 out of 12 subjects rated the SIN stimulation as more comfortable than the FM stimulation. Moreover, no stimulation segment was rated as “5—very comfortable”, which decreases the upper end of our custom scale. The rating “1—very uncomfortable” was used by three subjects, but only for the SIN stimulation condition. In order to exclude potential order effects which could confound our analysis, we calculated a mixed factor analysis of variance (ANOVA) with stimulation condition as a within-subject factor and the pseudo-randomized block order as a between-subjects factor. The ANOVA confirmed the main effect of stimulation condition (*F*_(1,10)_ = 9.918, *p* = 0.01, $\eta_{\text{p}}^{2}$ = 0.50). While there was no significant effect of block order (*F*_(1,10)_ = 0.738, *p* = 0.41, $\eta_{\text{p}}^{2}$ = 0.07), we found a significant interaction (*F*_(1,10)_ = 11.12, *p* < 0.01, $\eta_{\text{p}}^{2}$ = 0.53) with ratings for the SIN condition decreasing when presented after the FM condition, and ratings for the FM condition increasing when presented after the SIN condition. However, as our experiment was not designed to test for between subject effects, the comparisons including block order have a small sample size and should be regarded with caution. Potentially, the significant interaction could reflect a context effect with a comfortable stimulus becoming even more comfortable after an uncomfortable one and vice versa.

**Table 1:** Individual comfort ratings after the stimulation, averaged for the sinusoidal (SIN) and frequency modulated (FM) conditions, respectively.

Subject	1	2	3	4	5	6	7	8	9	10	11	12	Mean
SIN rating	3	3	3.5	1	3	2.5	2.5	2.5	3.5	1.5	3	1	2.5
FM rating	3	3	3	2	4	3	3	3	3.5	2	4	2	3
Comparison	FM rating > SIN rating → *t*_(11)_ = 3.19, *p* = 0.004												

*Higher values indicate better comfort*.

### SSVEPs

**Figure 3:**
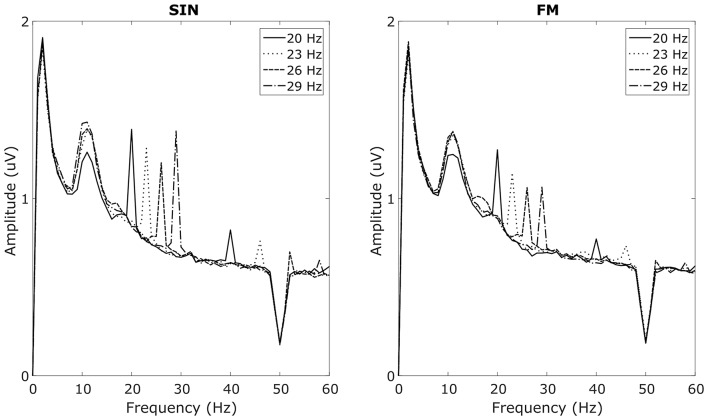
Average SSVEP spectra. Averaged over subjects with visible SSVEP peaks in the target frequencies and averaged over O1 and O2. Left plot shows responses to sinusoidal (SIN) stimulation, right plot shows responses to FM stimulation. Line styles corresponds to the four stimulation frequencies. For each style, two peaks at the corresponding stimulation frequency as well as at its first harmonic can be seen. The broad peak around 10 Hz reflects spontaneous alpha oscillations.

In the overt attention blocks, reliable SSVEP peaks in the SIN, as well as in the FM condition could be recorded in 9 out of 13 subjects with 1 s long analysis windows. These nine subjects will subsequently be referred to as responders. Spectra from the four non-responders are provided in Supplementary Figure S1. Averaged spectra from the two occipital electrodes, averaged over the responders can be seen in Figure 3. These spectra for the respective stimulation frequencies have clear peaks at the stimulation frequencies, as well as their first harmonics. No simultaneous power increases at the stimulation frequencies of the other LEDs can be seen. All of the responders had peaks in both, the SIN as well as the FM condition. Moreover, the non-responders had no visible peaks in either condition. Covert spatial attention shifts while fixating the central LED were not sufficient to evoke visible SSVEP peaks in any of the subjects.

**Figure 4:**
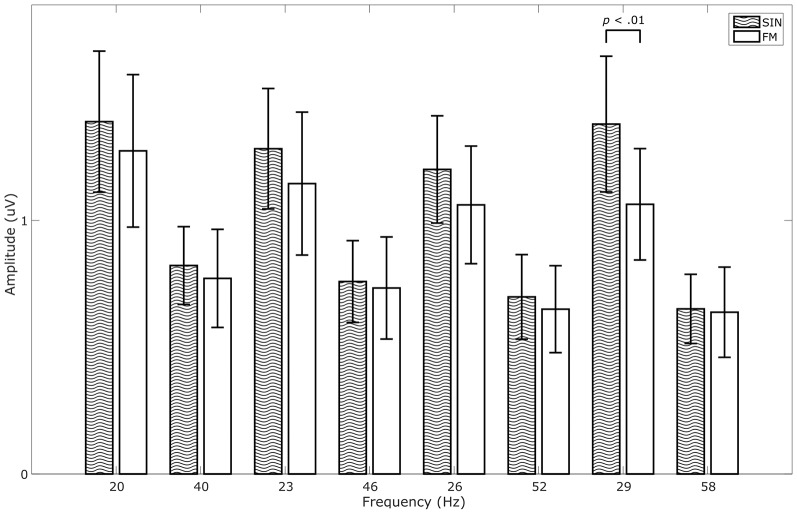
Comparison of SSVEP amplitudes across conditions. Data were averaged across 1 s epochs, across responders and across the occipital channels O1 and O2. Data for all stimulation frequencies as well as their first harmonics are shown. Error bars show standard deviations.

We calculated a repeated measures ANOVA with frequency and stimulation condition as factors. We found significant main effects for frequency (*F*_(7,56)_ = 16.504, *p* < 0.001, $\eta_{\text{p}}^{2}$ = 0.67), for stimulation condition (*F*_(1,8)_ = 6.985, *p* < 0.05, $\eta_{\text{p}}^{2}$ = 0.47) and a significant interaction between the two (*F*_(7,56)_ = 2.912, *p* < 0.05, $\eta_{\text{p}}^{2}$ = 0.27). Although average SIN-SSVEP amplitudes tend to be higher than the average FM-SSVEP amplitudes, a further statistical comparison of the individual amplitudes at the target spectral peaks with paired-sample *t*-tests across subjects only revealed a significant difference for 29 Hz (*t*_(8)_ = 4.16, *p* < 0.01; Figure 4). All other comparisons did not fall below the significance threshold of *p* < 0.05. This suggests that SSVEPS amplitudes are roughly comparable between SIN and FM stimulation, which replicates the results of Dreyer and Herrmann (2015).

### Classification

Offline classification of the filtered time-series data was done using CCA. For classification, we tested epoch lengths between 1 s and 11 s in steps of 1 s. Note that splitting the 11 s intervals led to different numbers of epochs available for the different epoch lengths. Per target LED, we obtained 110 1 s epochs, 50 2 s epochs, 30 3 s epochs, 20 4 s and 5 s epochs and 10 epochs for 6 s, 7 s, 8 s, 9 s, 10 s and 11 s. The classification accuracies reported below are averages over all four stimulation frequencies.

**Figure 5:**
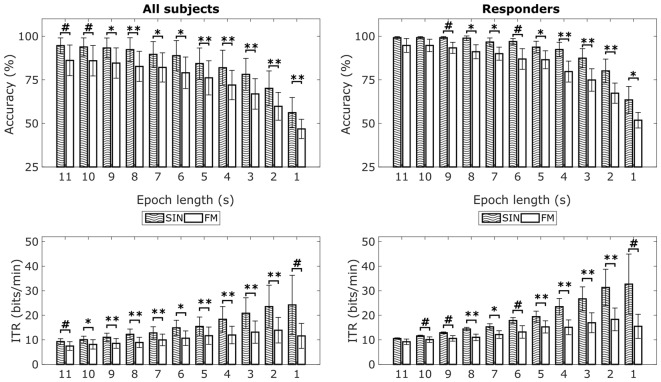
Mean classification accuracies and information transfer rates (ITRs) for FM and SIN stimulation with overt attention. Left column shows means over all subjects, right column shows means of responders only. The theoretical chance level with four simultaneously flickering LEDs is 25%. Significance indicators relate to paired-sample *t*-tests across subjects: ^#^*p* < 0.05, *p < 0.0045, **p < 0.001. *Relates to the Bonferroni corrected p-threshold for a 95% significance level (0.05/11 = 0.0045). Error bars show standard deviations.

Mean classification accuracies for the two conditions can be seen in Figure 5 (top row). The 1 s epochs lead to an average classification accuracy of 56% for the SIN stimuli (range: 33%–87%) vs. 47% for the FM stimuli (range: 29%–69%). Accuracies in both conditions continuously increase with increasing epoch lengths. Eleven second epochs lead to an average classification accuracy of 95% for the SIN stimuli (range: 73%–100%) vs. 86% for the FM stimuli (range: 45%–100%). Independent of epoch lengths, mean classification accuracies are clearly above the theoretical chance level which was at 25% for our four LED modulation frequencies. On a single subject level, only the four non-responding subjects show classification accuracies below 40% with 1 s epoch length. This is in accordance with the lack of a clear peak in the 1 s spectra. For longer epoch lengths we found increased accuracies in all subjects, including the non-responders. Three of the non-responders pass 40% with 2 s epoch length and one passes 40% accuracy with an epoch length of 7 s. This means, that even if no SSVEPs peaks are visible in 1 s spectra, we were still able to correctly classify the data from all subjects at a significant level by using longer time windows for classification. Therefore, we also tried using CCA to classify the data from the covert spatial attention blocks, which did neither result in visible SSVEP peaks. However, offline classification of this data showed less promising results. Independent of epoch length, no subject was classified with an accuracy over 35% in the first FM covert attention blocks. In the second FM covert attention blocks, we reached an accuracy of 40% for two subjects and epoch lengths of 10 s or more. In general, the FM stimulation with covert attention shifts only was not successful.

In addition to classification accuracies for the overt attention conditions, Figure 5 also shows the corresponding average ITRs for the different epoch lengths. For SIN, 1 s epochs lead to highest average ITR of 24.21 bits/min (range: 1.37–76.1 bits/min). For FM, 2 s epochs lead to the highest average ITR of 13.91 bits/min (range: 0.33–35.19 bits/min). Paired-sample *t*-tests revealed that accuracies as well as ITRs for most epoch lengths are on average higher for the SIN stimulation than for the FM stimulation. As indicated by the respective standard deviations and ranges, the inter-individual differences are quite large. We additionally analyzed the epoch length with maximal ITRs on a single-subject basis. Here, the best ITR is on average reached at 3.85 s for SIN stimulation and 3.46 s for FM stimulation. The corresponding *t*-test did not reveal a significant difference (*t*_(12)_ = 0.49, *p* = 0.63), so the epoch length needed to reach maximum ITR seems to be similar for both conditions.

## Discussion

The current study had four aims. First, we wanted to show that frequency-specific FM-SSVEPs can be reliably evoked using multiple LEDs simultaneously flickering at different FM frequencies. We were able to show FM-SSVEP peaks at 20 Hz, 23 Hz, 26 Hz and 29 Hz with a carrier frequency of 100 Hz. In addition to FM-SSVEP peaks at the fundamental frequencies, we found evoked spectral peaks at the first harmonic which can be useful for classification (Müller-Putz et al., 2005). Finding peaks at all fundamental frequencies is important because in our previous study we only showed FM-SSVEP peaks at 10 Hz which could have been caused by entrainment of the alpha rhythm (Dreyer and Herrmann, 2015). The results of the present study indicate that FM-SSVEPs allow for multi-class discrimination paradigms. Offering choices among several commands is essential for BCIs with real-world application like spellers. In principle, more than four FM frequencies could be implemented by using more LEDs. Thereby, more commands could be possible. Other studies have shown the suitability of SSVEP-BCIs with many more LEDs with closer spacing than we used here (Hwang et al., 2012). We believe that such systems could equally work with FM-SSVEPs.

Several studies using SSVEPs have reported users experiencing fatigue, annoyance or discomfort due to the flicker stimulation (Ortner et al., 2011; Lin et al., 2012; Cao et al., 2014). Therefore, our second aim was to show that FM-SSVEP stimulation would be more comfortable for the user. Our FM stimulation was based on a 100 Hz SIN carrier signal, which has been shown to lead to FM-SSVEPs that are less perceptible than SIN-SSVEPs (Dreyer and Herrmann, 2015). While this might not lead to reduced fatigue of the visual system, it should decrease the discomfort and thereby improve the user experience. Indeed, we found a significantly higher comfort rating for the FM stimulation condition. Eleven out of 12 subjects rated the FM stimulation as equally or more comfortable than the SIN stimulation and only three subjects rated it as slightly uncomfortable. We therefore suggest, that FM stimulation should be considered for future SSVEP-BCI applications, especially when designing communication tools for patients, for whom a comfortable user experience is particularly important.

Our third aim was to compare SSVEP amplitudes, offline classification accuracies, and resulting ITRs between SIN- and FM-SSVEPs. With respect to FM-SSVEP ITRs, the results are in the range of other studies that used LED stimuli. Reviewing five LED-based SSVEP-BCI studies with comparable numbers of commands (<10) Vialatte et al. (2010) report an ITR range of 3.2 bits/min–51.5 bits/min (mean = 19.5 bits/min). On average over subjects we obtained an ITR of 18.33 bits/min with FM stimulation and 2 s epochs lengths (responders only) which is comparable. In concordance with Dreyer and Herrmann (2015) we find that SSVEP amplitudes did not differ significantly between FM and SIN stimuli (except for the 29 Hz stimulus), when using paired-sampled *t*-tests. The amplitudes of the spectral stimulus peaks were lower for the FM than for the SIN stimulation (compare Figures 1A,D), which indicates that amplitude differences between the SIN and FM stimuli at the stimulation frequencies do not equally translate into power spectral differences between the respective SSVEPs. A supplementary ANOVA revealed a main effect of stimulation condition, which was however mainly driven by the large difference at 29 Hz. The effect sizes of the stimulation condition on SSVEPs amplitude ($\eta_{\text{p}}^{2}$ = 0.47) and comfort rating ($\eta_{\text{p}}^{2}$ = 0.50) are similar with a tendency towards a greater benefit on comfort when using FM stimuli. However, we found that classification accuracies and ITRs were overall lower for the FM condition, but the difference between classification accuracies decreases with longer analysis epochs when accuracies reach ceiling level. The improved comfort of the FM signal seems to go along with slightly decreased BCI performance. It is important to note, that rather than maximizing classification accuracies, our goal in this study was to provide a general proof of concept. In order to further mitigate differences in BCI performance one could potentially tweak the stimuli as well as the classification algorithms to bring both closer to ceiling level classification accuracy. During recent years, several approaches to improve CCA performance have been developed (Zhang et al., 2014; Chen et al., 2015) and these could be implemented for FM-SSVEP classification as well (see Nakanishi et al. (2015) for a direct comparison of different CCA-based approaches). Moreover, the LEDs we used for stimulation covered only around 0.57° of the visual field, which is quite small compared to stimuli used in other BCI studies. Ng et al. (2012) showed that larger stimuli result in increased SSVEP amplitudes and higher classification accuracies. They suggest that SSVEP-BCI stimuli should be larger than 2° visual angle which could easily be implemented with FM driving signals and should significantly improve classification accuracies. In addition, Reichert et al. (2013a) found that visual feedback can increase BCI performance. This would require online implementation of our approach.

Our last aim was to evoke FM-SSVEP with covert spatial attention shifts performed while subjects fixated a central LED. Such covert shifts could make a BCI system independent of eye movements. Covert visual attention variation has been shown to be suitable for BCI approaches (Zhang et al., 2010). However, the literature on studies showing working BCI systems based on covert attention shifts is sparse. Müller et al. (1998) showed SSVEP amplitude increases for spatially attended stimuli and Ordikhani-Seyedlar et al. (2014) showed increased harmonic responses with covert spatial attention shifts. Both could potentially be used as BCI features. Kelly et al. (2004) report a significant drop in classification accuracy with covert spatial attention compared to overt attention. Walter et al. (2012) directly compared overt and covert attention effects and suggest that overt attention allows for more reliable BCI systems, due to a decreased SSVEP amplitude modulation of covert attention. It can be assumed, that the sparse covert spatial attention BCI literature is a consequence of lower amplitudes of the modulatory effects leading to lower classification accuracies or even failing BCI systems. Similarly, our approach of FM stimulation with covert spatial attention did not evoke reliable SSVEP peaks and consequently, the classification failed. Several subjects reported difficulties understanding the task and not knowing what was expected from them which could be one reason for this negative result. In order to better clarify the task, we would train our subjects on a spatial attention paradigm, like reacting to random color-changes of the attended LED, in future studies. A second reason for the negative result could lie in our stimulus properties and setup. We used rather small stimuli which cause lower amplitudes (Ng et al., 2012) even when directly fixated (overt attention). Moreover, these already small stimuli fall onto the retinal periphery when only covert spatial attention is directed towards them. The smaller anatomical representation of peripheral locations in early visual areas may have led to even lower, undetectable in our case, SSVEP amplitudes for peripheral stimuli (Lin et al., 2012). A third reason could lie in the mechanisms of SSVEP generation. Two mechanisms have been proposed. On the one hand, it is conceivable that every single light flash evokes a VEP and repetitive flashes result in a superposition of as many VEPs as flashes were presented (Capilla et al., 2011). On the other hand, Notbohm et al. (2016) have shown that repetitive light flashes can entrain the human EEG alpha rhythm. Most probably, the latter mechanism of entrainment is at work with light flash frequencies close to the individual alpha frequency of subjects around 10 Hz. In contrast, the former mechanism of superposition may be at work when the frequency of light flashes is beyond the range of ongoing EEG rhythms. Since the EEG amplitude of alpha oscillations is on the order of some 10 μV while the amplitude of VEPs is in the order of only a few microvolts, SSVEPs elicited due to entrainment of the EEG alpha rhythm are likely to be of higher amplitude.

## Conclusion

FM-SSVEPs are suitable stimuli for BCIs and preferable in terms of user comfort. We were able to reliably evoke them in the 20–29 Hz range with four independently flickering LEDs. The benefit of FM stimulation is an increased comfort level when compared with traditional SIN stimulation. There is a tradeoff between higher comfort and slightly reduced classification performance which could supposedly be eliminated with an optimized stimuli design and a further optimized classification algorithm, specifically designed for FM-SSVEPs. The actual performance in an online BCI application remains to be tested in future research.

## Author Contributions

AMD: study design, manuscript preparation, data acquisition and analysis. CSH: study design, manuscript preparation and study supervision. JWR: study design, manuscript preparation and study supervision. All authors read and approved the final manuscript. CSH and JWR contributed equally.

## Conflict of Interest Statement

The authors declare that the research was conducted in the absence of any commercial or financial relationships that could be construed as a potential conflict of interest.

## Abbreviations

BCI: brain-computer interface
CCA: canonical correlation analysis
EEG: electroencephalography
FM: frequency modulated
ITR: information transfer rate
LED: light-emitting diode
PSDA: power spectral density analysis
SIN: sinusoidal
SNR: signal-to-noise ratio
SSVEP: steady-state visually evoked potential.
