# Supplementary material for: Tradeoff between User Experience and BCI Classification Accuracy with Frequency Modulated Steady-State Visual Evoked Potentials
Source: Front Hum Neurosci. 2017 Jul 26;11:391. doi: 10.3389/fnhum.2017.00391 (PMC5526890; doi:10.3389/fnhum.2017.00391)
Supplement: Supplementary file 1 [file Image_1.pdf]

Supplementary Material

Tradeoff between User Experience and BCI Classification Accuracy with Frequency Modulated Steady-State Visual Evoked Potentials

Alexander M. Dreyer*, Christoph S. Herrmann, Jochem W. Rieger

*** Correspondence:** Alexander Dreyer: alexander.dreyer@uni-oldenburg.de

1 Supplementary Figure

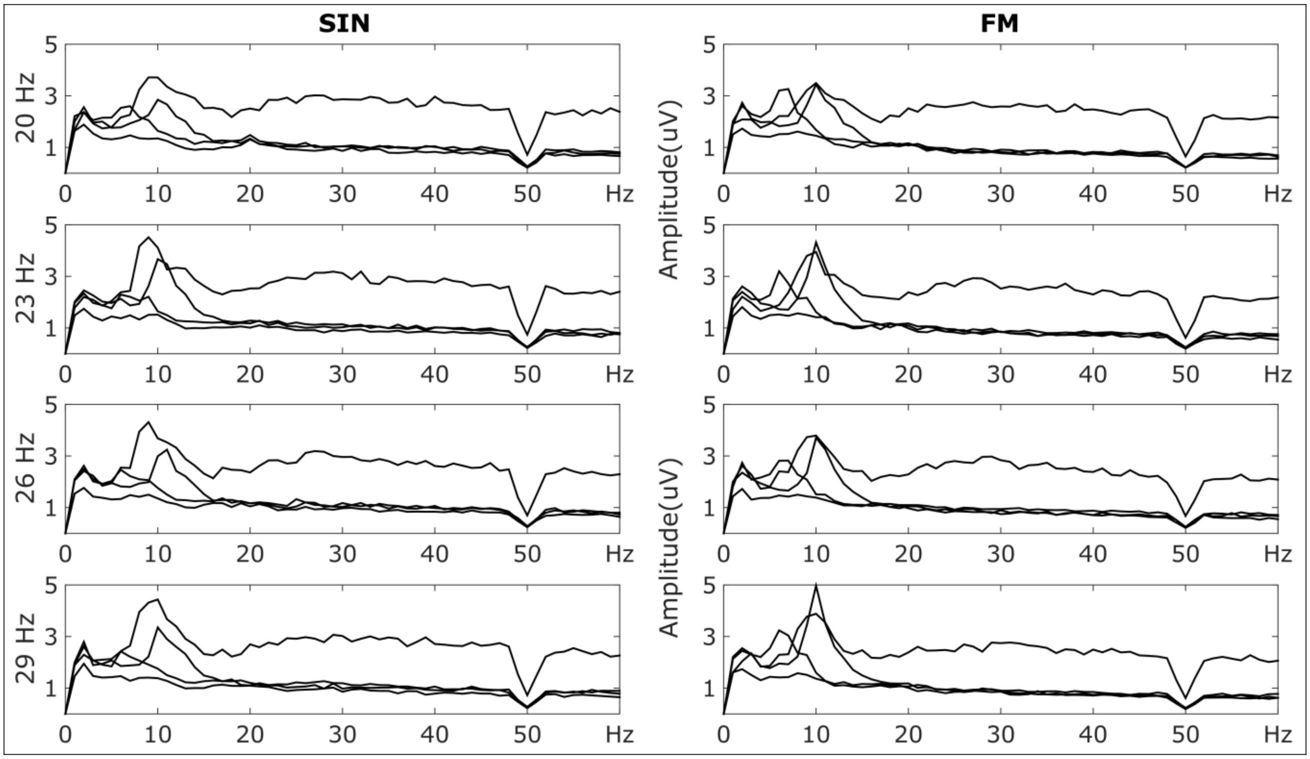

Supplementary Figure 1. Frequency spectra of the four non-responding subjects averaged over O1 & O2. Each subplot respectively shows four spectra (each line represents one subject) from one stimulation condition (SIN vs. FM in the columns) and one stimulation frequency (20Hz, 23Hz, 26Hz, 29Hz in the rows). As the stimulation did not evoke discernible SSVEP peaks, these subject were excluded from the SSVEP amplitude comparison between the stimulation conditions.
